# B7H3 in Gastrointestinal Tumors: Role in Immune Modulation and Cancer Progression: A Review of the Literature

**DOI:** 10.3390/cells14070530

**Published:** 2025-04-02

**Authors:** Sylwia Mielcarska, Anna Kot, Agnieszka Kula, Miriam Dawidowicz, Piotr Sobków, Daria Kłaczka, Dariusz Waniczek, Elżbieta Świętochowska

**Affiliations:** 1 Department of Medical and Molecular Biology, Faculty of Medical Sciences in Zabrze, Medical University of Silesia in Katowice, 41-800 Zabrze, Poland; s85876@365.sum.edu.pl (A.K.); s86196@365.sum.edu.pl (P.S.); s73707@365.sum.edu.pl (D.K.); 2 Department of Oncological Surgery, Faculty of Medical Sciences in Zabrze, Medical University of Silesia, 40-514 Katowice, Poland; d201070@365.sum.edu.pl (A.K.); d201069@365.sum.edu.pl (M.D.); dwaniczek@sum.edu.pl (D.W.)

**Keywords:** B7H3 (CD276), immune checkpoint, gastrointestinal cancers, immunotherapy

## Abstract

B7-H3 (CD276), a member of the B7 immune checkpoint family, plays a critical role in modulating immune responses and has emerged as a promising target in cancer therapy. It is highly expressed in various malignancies, where it promotes tumor evasion from T cell surveillance and contributes to cancer progression, metastasis, and therapeutic resistance, showing a correlation with the poor prognosis of patients. Although its receptors were not fully identified, B7-H3 signaling involves key intracellular pathways, including JAK/STAT, NF-κB, PI3K/Akt, and MAPK, driving processes crucial for supporting tumor growth such as cell proliferation, invasion, and apoptosis inhibition. Beyond immune modulation, B7-H3 influences cancer cell metabolism, angiogenesis, and epithelial-to-mesenchymal transition, further exacerbating tumor aggressiveness. The development of B7-H3-targeting therapies, including monoclonal antibodies, antibody–drug conjugates, and CAR-T cells, offers promising avenues for treatment. This review provides an up-to-date summary of the B7H3 mechanisms of action, putative receptors, and ongoing clinical trials evaluating therapies targeting B7H3, focusing on the molecule’s role in gastrointestinal tumors.

## 1. Introduction

The treatment of cancer has undergone significant changes over the past decade with the advent of therapies that promote anti-tumor immunity. A crucial aspect of this process is the balance between signals that stimulate and inhibit immune responses, allowing for an effective defense against pathogens and malignancies while maintaining self-tolerance to the antigens [1]. Immune checkpoints are crucial in controlling immune responses by delivering both costimulatory and coinhibitory signals, which can either enhance or suppress T-cell activity and regulate its duration. Tumor cells frequently overexpress negative immune checkpoints, which deliver coinhibitory signals that allow cancer cells to evade immune surveillance and reshape the tumor microenvironment into an immunosuppressive landscape. Consequently, blocking these immune checkpoints with pharmacological agents restores anti-tumor immunity and has become one of the primary strategies utilized in current immunotherapies [2].

Members of the B7 immune checkpoint family are proteins expressed on both immune and tumor cells within the microenvironment of various cancer types. B7 coinhibitory ligands, through interactions with their receptors, effectively inhibit the signals induced by the MHC-TCR complex between antigen-presenting cells (APCs) and T lymphocytes, ultimately leading to the apoptosis and anergy of T lymphocytes [3]. Conversely, interactions between CD28 and B7 costimulatory ligands promote the activation of antigen-specific T cells, upregulate cytokine expression, facilitate T-cell differentiation and expansion, and inhibit pro-apoptotic signals, thereby enhancing the immune response [4]. Beyond their crucial role in modulating immune responses, these proteins also contribute to cancer progression, invasion, metastasis, and drug vulnerability, independent of immune mechanisms. To date, ten members of the B7 family have been identified: B7-1 (CD80), B7-2 (CD86), B7-H1 (PD-L1), PD-L2 (PDCD1LG2, B7-DC), B7-H2 (ICOS-L, B7RP1), B7-H3, B7-H4 (B7x, Vtcn1, and B7S1), VISTA (B7-H5), B7-H6 (NCR3LG1), HHLA2 (B7-H7), and ILDR2 [5]. Notably, PD-1 and its ligands, PD-L1 and PD-L2, are the most extensively studied proteins within the B7 family and have significantly changed the landscape of cancer therapy. However, due to their limited expression in certain types of tumors, particularly those characterized as ‘immune cold’, and the resistance to the anti-PD-1/PD-L1 blockade, there is an urgent need for novel immune targets [6].

**Figure 1:**
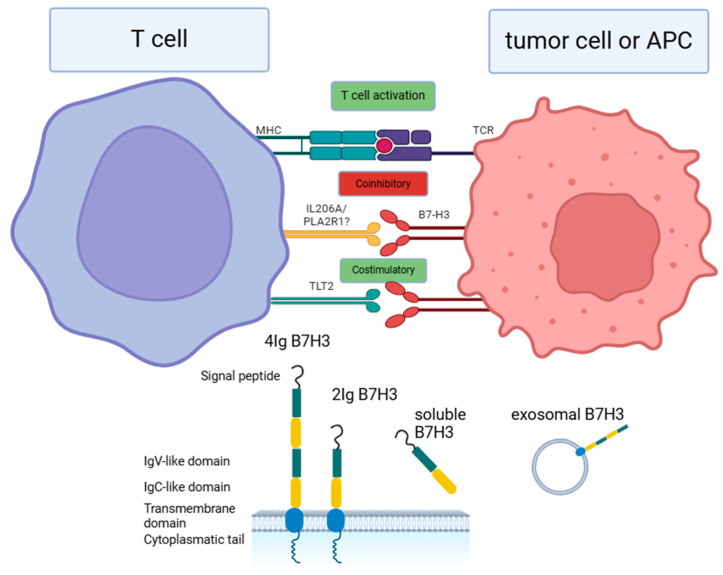
B7H3 structure and its putative receptors (https://BioRender.com/i17c845, accessed on 22 March 2025).

B7-H3 was first identified in 2001 as a costimulatory member of the B7 family of proteins; however, accumulating evidence over the years has highlighted its significant coinhibitory role in the regulation of immune responses [1,7]. In humans, the gene encoding B7-H3, also referred to as CD276, is located on chromosome 15 [8]. This type I transmembrane protein consists of 316 amino acids and exhibits a 27% amino-acid identity with B7-H1, 25% with B7-H2, 21% with B7-1, and 20% with B7-2 [7]. Human B7-H3 exists in two isoforms: 2IgB7-H3 (B7-H3 VC) and 4IgB7-H3 (B7-H3 VCVC), whereas the murine B7-H3 incorporates only one isoform (2IgB7-H3) (Figure 1) [7]. The 2IgB7-H3 isoform consists of single extracellular IgV-like and IgC-like domains, a transmembrane region, and a 45-amino-acid cytoplasmic tail [9]. In contrast, the 4IgB7-H3 isoform contains two identical pairs of IgV-like and IgC-like domains (four Ig-like domains in total) and is predominantly expressed on the surface of human mononuclear and tumor cells [9]. Notably, the FG loop of the IgV domain has been identified as critical for inhibiting the proliferation of naïve T cells in vitro and in murine models [10]. Mutations within the FG loop resulting in a marked reduction in B7-H3’s inhibitory activity suggest that this structural feature may represent a promising therapeutic target for strategies aimed at B7-H3 inhibition. In addition to the 2IgB7-H3 and 4IgB7-H3 isoforms present on the cell surface, a soluble form of B7-H3 (sB7-H3) has been identified in human serum and extracellular vesicles. This soluble variant is likely derived from the proteolytic cleavage of the 2IgB7-H3 isoform, a process facilitated by matrix metalloproteinases (MMPs), or it may result from alternative splicing of B7-H3 mRNA [11]. Although the complete characterization of B7-H3 receptors remains elusive—thereby complicating efforts to target it with antineoplastic agents—B7-H3 has been shown to bind to several receptors, including TREM family member TLT-2, PLA2R1, and IL20RA [12,13,14]. B7-H3 expression is observed at low levels across various human tissues, indicating that posttranscriptional and posttranslational regulatory mechanisms significantly influence its expression [15]. The high prevalence of the B7H3 protein in numerous malignancies—including lung cancer, esophageal squamous cell carcinoma (ESCC), gastric cancer, pancreatic cancer, colorectal cancer, liver cancer, breast cancer, brain tumors, and prostate cancer—further underscores its potential clinical relevance, as the upregulation of B7H3 was found to be associated with tumor cell proliferation, metastasis, treatment resistance, and ultimately, poor prognosis. The expression patterns of B7H3 exhibit predominantly cytoplasmic and membrane localization, with occasional nuclear presence [16]. The pronounced disparity between B7-H3 levels in normal and neoplastic tissues positions this protein as an attractive therapeutic target, as its selective targeting may yield cancer-specific cytotoxicity while sparing normal tissues. This review aims to elucidate the role of B7H3 in gastrointestinal tumors, due to the still unsatisfactory results of currently available immunotherapeutic options for this group of patients, resulting in the need for the identification of novel, promising immune checkpoints. The vast subject of the B7H3 function in other solid tumors goes beyond this article, and it was not covered in the review.

## 2. Receptors for B7-H3

While considering receptors for B7-H3, it is crucial to distinguish between forward and backward signaling in relation to this molecule. Forward signaling refers to the process whereby B7-H3 binds to unknown partners on immune cells, thereby modulating immunological responses. Conversely, backward signaling designates the interaction where B7-H3 functions predominantly as a receptor on tumor cells rather than as a ligand [17,18]. To date, the precise receptors for B7-H3 have not been definitively identified; however, several candidate molecules have been proposed, including triggering receptors expressed on myeloid cells (TREM)-like transcript 2 (TLT-2, TREML2), interleukin-20 receptor subunit alpha (IL20RA), phospholipase A2 receptor 1 (PLA2R1), and other potential partners such as 4-1BB and AAMP [19,20]. Notably, findings regarding the functions of these putative receptors remain ambiguous and contradictory [17,21].

Among these candidates, TLT-2 is the most extensively investigated potential binding partner of B7-H3. B7-H3 has been implicated as an immune costimulator, promoting IFN-γ production and enhancing CD8 T-cell functionality via TLT-2 [12]. Initial investigations suggested a direct interaction between TLT-2 expressed on CD8 T cells and B7-H3, thereby augmenting cytotoxic activity [22]. Conversely, in monocytes, TLT-2 appears to have an inhibitory effect on Th1 differentiation and suppresses immune responses to tuberculosis, likely through the activation of the JAK/STAT3 signaling pathway and subsequent IL-6 secretion [23]. This suggests that TLT-2 may exert opposing effects on immune regulation depending on the cellular context, which may explain the contradictory roles of B7-H3 in modulating immunological processes. However, the validity of the B7-H3-TLT-2 interaction has been questioned by multiple authors who found the existing evidence insufficient [24,25]. Consequently, the costimulatory roles attributed to B7-H3 may be contingent upon yet-to-be-identified binding partners, leaving this area of research poorly understood [24].

Another candidate receptor for B7-H3 is PLA2R1, a member of the mannose receptor family, whose expression is reportedly diminished across various tumor types. It has been proposed as a tumor suppressor that promotes cellular senescence through the induction of reactive oxygen species in mitochondria, the repression of PARP1 expression, and the activation of the p53 pathway [26]. Additionally, PLA2R1 has been linked to the inhibition of cell neoplasia via estrogen-related receptor α1 and JAK2 signaling cascades [27]. B7-H3-PLA2R1 interactions were reported by Cao et al. using the Receptor Display In Membranes Interaction Screen (RDIMIS) platform [28]; these findings warrant further verification in subsequent investigations to better elucidate the role of this interaction in tumorigenesis.

IL20RA represents another potential target for B7-H3. IL20RA is known to transmit signals from IL19, IL20, and IL24 through interactions with IL20RB, leading to the activation of JAK1/STAT3 pathways. These signaling events can elicit both tumor-supporting and tumor-suppressing effects [29]. Utilizing the Conditioned Media AlphaScreen platform, Husain et al. identified IL20RA as a binding partner for B7-H3, which was confirmed by Cao et al. [14,28]. Nevertheless, further investigation is required to elucidate the significance of the B7-H3-IL20RA interaction in the context of tumorigenesis.

B7-H3 has also been proposed to bind to the TNF receptor 4-1BB (TNFRSF9, CD137), a member of the tumor necrosis factor superfamily that is expressed in activated T cells, B cells, NK cells, and dendritic cells, as well as in various malignancies [30]. Its primary binding partner, 4-1BBL (TNFSF9), is a type II membrane protein within the TNF ligand superfamily [31]. 4-1BB functions as a costimulatory molecule, playing a pivotal role in eliciting immune responses, primarily through the activation of CD8 T cells, thus rendering it an attractive target in immunotherapeutic strategies [30]. The interactions between B7-H3 and 4-1BB remain a subject of contention within the field. Research conducted by Ma et al. indicates that the knockdown of B7-H3 in nasopharyngeal carcinoma results in an increased proportion of 4-1BB+CD8+ tumor-infiltrating lymphocytes (TILs). This observation suggests that the expression of B7-H3 may lead to the downregulation of 4-1BB, thereby inhibiting its anti-tumor efficacy [21]. Furthermore, the authors hypothesize that the B7-H3 and 4-1BB interaction could contribute to a reduction in the apoptosis of tumor cells; however, these findings necessitate further validation to establish their definitive impact [21].

Finally, the angio-associated migratory cell protein (AAMP) was identified as another significant interactor for B7H3. AAMP is predominantly expressed in the cytoplasm and membrane of vascular endothelial cells and is characterized by a WD40 domain and immunoglobulin-like domains [32]. AAMP plays a crucial role in angiogenesis and the migration of both endothelial and cancer cells [32,33]. Notably, AAMP has been implicated in interactions with thromboxane A2 receptors and RhoA signaling pathways [32]. Ciprut et al. demonstrated that AAMP may serve as a potential binding partner for B7-H3 on natural killer (NK) cells. The authors posited that the interaction between AAMP and B7-H3 within human T lymphocyte (Jurkat) cells partially mediates the antiproliferative effects attributed to B7-H3 [34]. However, it is imperative to conduct further investigations to elucidate the binding dynamics between B7-H3 and AAMP.

In summary, the receptors for B7-H3 remain to be thoroughly defined, and existing findings are limited in their scope. A more comprehensive exploration of the relationships between B7-H3 and other membrane-associated molecules on both immune and cancer cells will be instrumental in enhancing our understanding of B7-H3’s functional role, potentially informing the development of novel therapeutic strategies.

## 3. Signaling Pathways

B7-H3 plays a pivotal role in modulating tumor cell metabolism through critical intracellular signaling pathways. Research has demonstrated that B7-H3 exerts its biological effects primarily by activating key signaling cascades, including the JAK/STAT, NF-κB, PI3K/Akt, Ras/Raf/MEK/MAPK, and NRF2/ROS pathways. These pathways are integral in regulating essential cellular processes such as growth, proliferation, differentiation, apoptosis, invasion, and metastasis [35,36,37].

### 3.1. JAK2/STAT3 Pathway

The Janus kinase 2/signal transducer and the activator of the transcription 3 (JAK2/STAT3) pathway facilitates the transduction of signals from the extracellular environment to the intracellular compartment following the binding of immune molecules and growth factors to their specific receptors on the cell membrane. The JAK2/STAT3 axis exerts control over a wide array of cellular processes, including proliferation, differentiation, and apoptosis. The dysregulation of this pathway is implicated in oncogenesis, tumor angiogenesis, and metastasis across multiple malignancies [38]. In colorectal cancer (CRC) cell lines, B7-H3 has been shown to enhance resistance to apoptosis through the upregulation of the JAK2/STAT3 signaling pathway, resulting in an increased expression of anti-apoptotic proteins such as Bcl-2 and Bcl-xL [36]. In ovarian cancer, the B7-H3 activation of JAK2/STAT3 was shown to inhibit apoptosis, promoting the proliferation, migration, and invasion of cancer cells [39].

Additionally, this signaling pathway has been identified as a mediator of the enhanced migration and invasion of cancer cells through the stimulation of matrix metalloproteinase-9 (MMP-9) production [40]. In breast cancer, the JAK2/STAT3 axis is involved in the B7-H3-mediated reduction in cancer cell sensitivity to Paclitaxel [41]. Notably, the silencing of B7-H3 expression led to the decreased phosphorylation of STAT3, reduced levels of downstream proteins Mcl-1 and Survivin, and an improved response to Paclitaxel treatment in murine models. Similarly, resistance to gemcitabine in pancreatic cancer cell lines has been linked to B7-H3 and was correlated with elevated levels of Survivin [42]. In various cancers, including malignant glioma, gastric cancer, salivary adenoid cystic carcinoma, and hepatocellular carcinoma (HCC), B7-H3 promotes epithelial-to-mesenchymal transition (EMT) via the activation of the JAK2/STAT3/Slug signaling pathway [43,44,45].

### 3.2. TLR4/NF-κB Pathway

The Toll-like receptor 4 (TLR-4), a member of the pattern recognition receptors (PRRs), plays a significant role in tumorigenesis associated with chronic inflammation. TLR-4 is activated in various neoplasms, contributing to protumoral activity by upregulating nuclear factor kappa B (NF-κB) transcription factors [46]. NF-κB transcription factors are crucial for regulating immune and inflammatory responses and are activated in response to foreign antigens and tissue injury. Beyond their physiological roles, the NF-κB pathway is significantly implicated in mediating protumoral effects, including the enhanced survival and proliferation of cancer cells [47]. In lung cancer, B7-H3 inhibits apoptosis in immunosuppressive tumor-associated macrophages (TAMs) under hypoxic conditions by enhancing NF-κB signaling; conversely, the knockdown of B7-H3 expression resulted in the increased apoptosis of TAMs. The expression of B7-H3 in macrophages has been shown to be regulated by miR-29-3p through exosomal mechanisms. However, in the tumor microenvironment, this regulatory mechanism is often disrupted, leading to increased anti-apoptotic signaling [48]. In colorectal cancer cell lines, NF-κB signaling has been implicated in promoting B7-H3-induced tumor angiogenesis by upregulating vascular endothelial growth factor A (VEGFA), while silencing B7-H3 resulted in the inhibition of NF-κB phosphorylation [35]. In pancreatic cancer cells, soluble B7-H3 was found to induce the production of interleukin-8 (IL-8) and VEGF-A by upregulating TLR4 and NF-κB signaling pathways. Furthermore, the TLR4/NF-κB axis has been associated with the enhanced invasion and metastasis of pancreatic cancer cells [49].

### 3.3. PI3K/AKT Pathway

The phosphoinositide 3-kinase/AKT (PI3K/AKT) signaling pathway is a critical downstream pathway frequently activated by various protumoral molecules. The activation of this pathway significantly contributes to tumor cell proliferation, survival, invasion, and metabolic processes through the upregulation of downstream effector proteins. In colorectal cancer cell lines, the PI3K/AKT signaling pathway, along with its downstream effector SMAD1, plays a crucial role in mediating B7-H3-induced EMT. This process leads to the disruption of epithelial cell polarity and adhesion, simultaneously enhancing the motility and invasiveness of cancer cells. The involvement of B7-H3 in this mechanism is supported by its effects on classical EMT markers, which include the downregulation of E-cadherin and β-catenin expression, along with the upregulation of N-cadherin and Vimentin levels [50]. In bladder cancer, the PI3K/Akt/STAT3 signaling pathway, which induces MMP-2 and MMP-9 expression, is crucial for enhancing the migration and invasion of cancer cells promoted by B7-H3 [51].

### 3.4. Ras/Raf/MEK/MAPK

Mitogen-activated protein kinase (MAPK) pathways play essential roles in the regulation of cell proliferation, differentiation, survival, apoptosis, and metabolism in normal cells. However, alterations in these pathways are well-recognized contributors to cancer growth and progression. The MAPK signaling network is complex and comprises four main branches, each characterized by its respective MAPK effectors: ERK1/2, ERK5, JNKs, and p38 MAPK [52]. In melanoma cells, p38 MAPK has been shown to mediate B7H3-induced resistance to chemotherapeutic agents such as dacarbazine and cisplatin. The inhibition of B7H3 expression enhances the sensitivity of tumor cells to these chemotherapeutics, correlating with a decrease in the phosphorylation level of the MAPK downstream effector, p38 [53]. In breast cancer cells, B7H3 promotes the population of cancer stem cells by binding to the major vault protein (MVP), which subsequently induces MEK through enhancing the interaction between B-RAF and MEK. The stimulation of the Raf/MEK/ERK pathway in breast cancer has been linked to B7H3-promoted lung metastasis [54].

### 3.5. NRF2/ROS

The primary function of the NRF2 signaling pathway is to protect cells from electrophilic and oxidative stress by inducing the expression of cytoprotective and antioxidative proteins, including SOD1, SOD2, and PRX3 [55]. The downregulation of the NRF2/ROS pathway leads to metabolic reprogramming in tumor cells, resulting in enhanced anaerobic glycolysis. This shift in cancer cell metabolism increases glucose turnover regardless of oxygen availability in the tumor microenvironment. In breast cancer cells, B7H3 has been reported to inhibit NRF2 and its downstream antioxidant proteins SOD1, SOD2, and SOD3. This inhibition increases the stability of HIF-1 alpha, thereby facilitating anaerobic glycolysis through the upregulation of glycolytic enzymes LDHA and PDK1, and by reducing pyruvate intake in the TCA cycle [56].

## 4. Immune Functions of B7-H3 in Gastrointestinal Tumors

The immunological role of B7-H3 in tumorigenesis has been the subject of extensive investigation. This protein has been detected across various cancer types, prominently expressed on diverse immune cell populations, as well as on tumor cells, within the tumor vasculature, and in carcinoma-associated fibroblasts (CAFs), thereby influencing the immunological landscape in malignancies [57,58]. Initially, B7-H3 was characterized primarily as a costimulatory molecule believed to enhance T-cell function and proliferation through TLT-2 [12]. Conversely, research by Leitner et al. provided no evidence supporting the B7-H3-TLT-2 interaction, instead characterizing B7-H3 as a potential inhibitor of T-cell function [25]. Still, the mechanisms governing the B7-H3-mediated modulation of immune responses remain poorly understood. Moreover, despite the growing number of studies, information on how the molecule regulates the immune landscape in gastrointestinal tumors is limited.

### 4.1. B7-H3 in the Regulation of TILs and Cytokine Secretion

While the increasing number of research studies emphasize the coinhibitory role of B7-H3 in the context of tumor-infiltrating lymphocytes (TILs) and cytokine regulation, its exact influence on immune processes remains controversial. Various studies have documented elevated B7-H3 expression in immune-cold tumors, alongside a negative correlation between B7-H3 expression and CD8 T-cell infiltrations [59,60]. Immune-cold tumors are characterized by reduced T-cell infiltrates, a diminished capacity to elicit immune responses, and a tumor microenvironment (TME) exhibiting immunosuppressive properties that hinder T-cell-mediated anti-cancer cytotoxicity [61]. These tumors also demonstrate limited responsiveness to immune checkpoint blockade (ICB) therapies [62]. A comprehensive analysis involving 156,791 samples from 50 types of malignancies has revealed a correlation between high B7-H3 expression and lower frequencies of CD8+ T cells across various tumor types [63]. Several studies have demonstrated that B7-H3 depletion leads to increased intratumoral frequencies of CD8+ and CD4+ T lymphocytes, although the influence of B7-H3 knockdown on CD4 T-cell infiltrates presents a more ambiguous picture [59,64]. In the murine models of induced esophageal squamous cell carcinoma (ESCC) with whole-body B7-H3 knockout, enhanced densities of CD8+ TILs were observed, while CD4 T cell density remained comparable to wild-type controls, a finding that was similarly reflected in models with epithelial B7-H3 silencing [59]. Intriguingly, research by Shen et al. identified a negative correlation between B7-H3 expression and immune infiltrations, as well as the majority of chemokines [65], while a positive relationship between cytoplasmic B7-H3 and CD8+ T cells was documented by Cattaneo and colleagues [66]. Furthermore, B7-H3 expression in colorectal cancer (CRC) was linked to more pronounced CD45RO+ infiltrations, which are associated with poorer prognosis and greater disease aggressiveness. CD45RO+ T cells play a critical role in regulating both T and B-cell functions, modulating immune responses, and potentially influencing the cancer phenotype through multiple pathways [67]. These findings suggest that the effects of B7-H3 on immune infiltrates may be contingent upon specific cancer types or additional influencing factors, indicating a need for a detailed examination of this complex regulation.

The effects exerted by B7-H3 on regulatory T cells (Tregs) are contentious and likely depend on cancer type. Tregs, a specialized subset of CD4+ T cells, are integral in maintaining immune tolerance and exerting immunosuppressive functions [68]. Numerous studies have failed to establish a significant association between B7-H3 expression and the infiltration of regulatory T cells, with some even indicating a negative correlation with these regulatory populations occurring in pancreatic ductal adenocarcinoma [66,69,70]. In clear cell renal cell carcinoma (ccRCC), however, B7-H3 was positively associated with FOXP3+ Tregs. Moreover, Inamura et al. suggested that the protumor role of the molecule in ccRCC may be facilitated through the regulatory T-cells [57].

Furthermore, B7-H3’s regulatory functions extend to various cytokine expressions, encompassing both pro-inflammatory and anti-inflammatory pathways. Research by Ma and colleagues proposed that B7-H3 could engage with 4-1BB, a tumor necrosis factor (TNF) receptor found on TILs, thereby influencing T-cell cytotoxicity. The inhibition of 4-1BB was shown to bolster CD8 T-cell cytotoxic functions, and knocking down B7-H3 increased the serum levels of IFN-γ and TNF-α in xenograft models [21]. Contrastingly, a study by Meng et al. concluded that B7-H3 was capable of enhancing TNF-α production, with B7-H3 silencing leading to reduced levels of TNF-α, IL-2, IL-4, and IFN-γ, while TNF-α itself could upregulate B7-H3 expression in colorectal carcinoma [71]. This interplay indicates a complex regulatory axis involving TNF-α, NF-κB signaling, and B7-H3, which could contribute to immune evasion in CRC. Specifically, B7-H3 upregulation was shown to occur via miR-34a-dependent inhibition of SIRT1, leading to the acetylation of the NF-κB subunit p65 (RelA). In another line of investigation, Peuker et al. highlighted that IL-6 could enhance B7-H3 expression in CRC through STAT3 signaling, implicating myeloid calcineurin—activated by microbiota—in mediating IL-6 production, which promotes CD276 expression and suppresses anti-tumor responses [72].

Additionally, the elevated expression of B7-H3 has been linked to enhanced TGF-β signaling pathways. Tumor Growth Factor-β (TGF-β) functions as an anti-inflammatory cytokine, facilitating proliferative processes during carcinogenesis and potentially augmenting the invasive capabilities of cancer cells [63]. Zhou and colleagues conducted a comprehensive investigation into the interplay between B7-H3 and TGF-β1, revealing that the upregulation of TGF-β1 via the SMAD3 and SMAD4 signaling proteins resulted in an increased expression of miR-155 in colorectal cancer. Notably, miR-155, which is often dysregulated in colorectal carcinoma (CRC), inhibits the transcription factor CEBPB, leading to the downregulation of miR-143 and the subsequent upregulation of its target genes, B7-H3 and B7-H4, in cancer cells [73]. At the same time, the overexpression of B7-H3 was found to diminish the secretion of key cytokines such as IL-4, IL-6, IL-17, TGF-β1, and TNF-α in HCT-116 cells, thereby establishing a negative feedback loop in the autocrine secretion mechanisms of CRC [73]. Conversely, B7-H3 upregulation was observed to promote the secretion of IL-2, IL-6, IL-17, and TGF-β1, while concurrently leading to reduced IFN-γ production in T-cells within the tumor microenvironment (TME) [73]. This differential regulation of autocrine signaling in tumor cells and cytokine production in immune cells may provide insights into the conflicting findings regarding CD276 and its impact on cytokine profiles as reported in various studies. Importantly, B7-H3 not only plays a crucial role in orchestrating immunological responses within the cancer milieu but is also subject to modulation by numerous immunological signals, thereby creating a nuanced and intricate relationship between the immune landscape and B7-H3 expression in tumors.

### 4.2. B7-H3 in the Regulation of Other Immune Cells

Several studies have established that the elevated expression of B7-H3 influences M1 and M2 macrophage infiltrations. M2 macrophages are characterized by an alternative activation state that fosters immunosuppressive activity and contributes to tumorigenesis. In contrast, M1 macrophages are primarily associated with pro-inflammatory responses and anti-tumor activity [74]. In ovarian cancer, B7-H3 was shown to take part in the differentiation and recruitment of M2 macrophages [60]. On the other hand, findings by Miller et al., in their analysis of 156,791 samples, indicated that high B7-H3 expression was associated with increased fractions of pro-inflammatory M1 macrophages, with minimal differences observed in M2 macrophage infiltrations across groups differentiated by B7-H3 expression levels [63]. Nevertheless, few data exist regarding the relationship between the molecule and macrophages in gastrointestinal tumors, and the topic could be expanded in future studies.

B7-H3 also modulates the activity of natural killer (NK) cells. Pathania and colleagues found that targeting the molecule can augment NK cell cytotoxicity in neuroblastoma (NB), explaining the possible mechanism of B7-H3-mediated immune evasion in NB [75]. Additionally, research by Xiong and colleagues observed increased NK cell proportions in immune infiltrates following B7-H3 silencing in mice with induced esophageal squamous cell carcinoma (ESCC). Their findings suggest an alternative regulatory mechanism wherein B7-H3 upregulation enhances the formation of neutrophil extracellular traps (NETs) through the activation of CXCL1–CXCR2 signaling. Given that NETs are capable of suppressing NK cell functions, elevated B7-H3 expression leads to a reduction in NK cell numbers, thereby facilitating tumorigenesis and disease progression. Moreover, B7-H3 knockdown resulted in decreased CXCL1 levels, inhibited NET formation, and increased NK cell infiltrations. B7-H3 silencing also correlated with reduced neutrophil percentages in immune infiltrates, alongside the downregulation of PI3K–Akt signaling pathways [76]. Additionally, Li et al. investigated B7-H3 expression in neutrophils within gastric cancer contexts, observing a correlation with poor patient prognosis. This upregulation was found to be enhanced by neutrophil activation through tumor-derived GM-CSF and occurred via the JAK-STAT3 signaling pathway [76].

Myeloid-derived suppressor cells (MDSCs) represent another immune cell subset influenced by B7-H3 expression. MDSCs, often present in chronic inflammatory conditions and advanced malignancies, exert immunosuppressive effects on T-cells and contribute to cancer progression [77]. In head and neck squamous cell carcinoma, a positive correlation was identified between B7-H3 and the MDSC markers CD11b and CD33 [70]. Collectively, the available reports picture B7-H3 as a multifaceted regulator of immune cell activity, contributing to altered defense mechanisms against tumorigenesis. Still, very few authors tackle the topic of B7-H3 influence on the immune microenvironment in gastrointestinal cancers. Further research is therefore crucial for the development of novel, effective therapeutic strategies for patients suffering from such malignancies.

### 4.3. Other Immune Processes Regulated by B7-H3

The role of B7-H3 in the modulation of immune responses within neoplasms extends beyond the previously discussed phenomena. This molecule is implicated in a multitude of processes, including the regulation of human leukocyte antigen (HLA) expression. Notably, the knockout of B7-H3 in tumor cells resulted in an upregulation of major histocompatibility complex class II (MHC-II) expression, as reported by Liu et al. [64]. Conversely, Cattaneo and colleagues did not replicate these findings; however, they did establish a positive correlation between B7-H3 and HLA class I expression specifically in pancreatic ductal adenocarcinoma (PDAC) [66]. HLA class I molecules facilitate critical interactions between neoplastic cells and the host immune system by presenting peptides derived from tumor antigens to cytotoxic T lymphocytes, thereby promoting the destruction of malignant cells [78]. Deficiencies in this process are commonly observed across various cancer types. In PDAC, the aberration of HLA class I functionality may correlate with tumor progression; defective HLA class I was identified in the majority of analyzed PDAC specimens. There was a positive correlation between the expression levels of HLA-A and cytoplasmatic but not membranous B7-H3 (*p* = 0.006), and similarly, higher HLA-B and HLA-C mRNA levels corresponded to elevated CD276 mRNA expression (*p* = 0.002 and *p* = 0.032, respectively). The authors hypothesized that p65 (RelA), a subunit of the NF-κB transcription factor, may facilitate the expression of both B7-H3 and HLA class I. Interestingly, the beneficial effects of functional HLA class I in PDAC were undermined by B7-H3 expression. In patients exhibiting high membranous B7-H3 expression, defective HLA-A/B/C or the low infiltration of CD8 T cells did not correlate with diminished survival outcomes, while such an association was present in cohorts with low B7-H3 expression. This suggests that B7-H3 plays a pivotal role in the suppression of anti-tumor immune responses within PDAC. Moreover, high levels of membranous B7-H3 expression were associated with decreased survival exclusively in patients demonstrating positive HLA class I expression, while no such correlation was observed in those with defective HLA-A/B/C. These findings indicate that B7-H3-targeting therapeutic strategies may be particularly advantageous for patients with positive HLA class I expression or could be synergistically combined with interventions aimed at upregulating HLA class I in PDAC. Further investigation into the intricate relationships between B7-H3 and HLA is warranted, as this knowledge could significantly contribute to the advancement of more effective anti-cancer therapies [66]. As demonstrated, B7-H3 exerts multifaceted influences on immune modulation in cancer, primarily functioning in an immuno-inhibitory capacity and fostering immune evasion across a range of malignancies. Nevertheless, the underlying mechanisms governing these roles remain complex and inadequately understood. A deeper elucidation of the pathways through which B7-H3 operates in various cancer types is essential for the development of robust therapeutic strategies in the future.

The effect of B7-H3 upregulation on immune responses in gastrointestinal tumors is summarized in Table 1.

## 5. Non-Immune Functions of B7-H3 in Tumorigenesis

**Figure 2:**
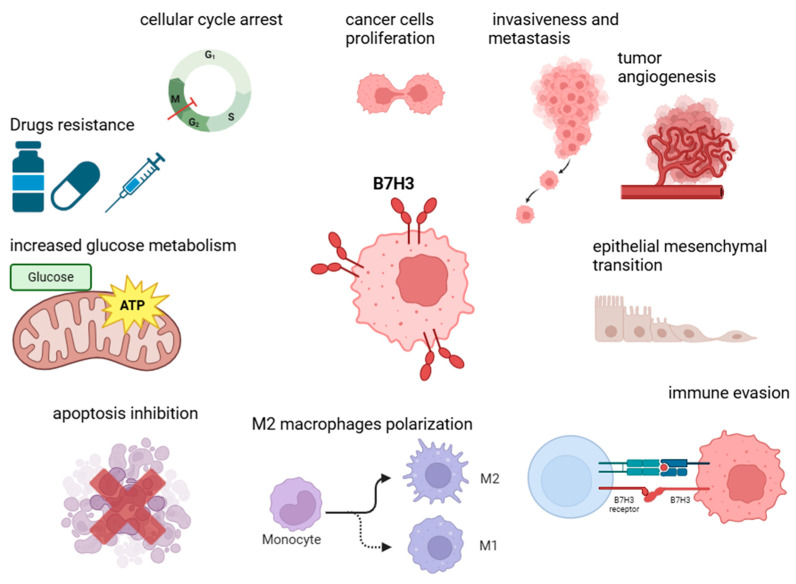
Protumoral effects induced by B7H3 in gastrointestinal malignancies (https://BioRender.com/v45f5hl, accessed on 22 March 2025).

In addition to its established role in modulating immunological responses, B7-H3 exhibits a plethora of non-immune functions that could significantly contribute to carcinogenesis, tumor progression, and the manifestation of more aggressive cancer phenotypes (Figure 2). Accumulating evidence indicates that B7-H3 is involved in several key cellular processes, including the proliferation, invasion, and migration of cancer cells, as well as angiogenesis and the development of drug resistance across various malignancies. A recent review has comprehensively addressed the non-immune mechanisms of B7-H3 in tumorigenesis [90].

### 5.1. Proliferation, Invasiveness, Migration, and Epithelial–Mesenchymal Transition (EMT)

B7-H3 has been shown to induce epithelial–mesenchymal transition (EMT) in various malignancies through the regulation of numerous signaling pathways, thereby promoting metastasis and contributing to a more aggressive disease phenotype [39,54]. Such protumorigenic effects have been documented in gastric cancer, where B7-H3 modulated the Jak2/STAT3 signaling pathway [44]. Moreover, B7-H3 influenced EMT processes in hepatocellular carcinoma by upregulating matrix metalloproteinases MMP-2 and MMP-9 or downregulating E-cadherin levels [91]. in head and neck squamous cell carcinoma (HNSCC), B7-H3 has been implicated in driving the invasion and metastasis of cancer stem cells, potentially through the activation of the AP-1 transcription factor [59]. Similarly, in pancreatic ductal adenocarcinoma (PDAC), the BRD4/B7-H3 axis regulated TLR4 expression, thereby enhancing the aggressive phenotype of the cancer [46]. However, a study by Sun et al. revealed that in a gastric cancer cell line, the increased expression of B7-H3 was associated with enhanced cell adhesion compared to a B7-H3-low-expression group. Moreover, treatment with exogenous fibronectin resulted in augmented cell adhesion in both groups, with a notable increase observed in the B7-H3-high group. These findings suggest that B7-H3 may engage in interactions with fibronectin to promote cell adhesion in gastric cancer [92]. In another study employing differentially expressed gene (DEG) analyses, evidence emerged that B7-H3 may participate in cytoskeletal motor activity and microtubule binding. Notably, B7-H3 expression showed a positive correlation with LIM domain kinase 1 (LIMK1) expression in colorectal cancer (CRC) tissue, revealing that B7-H3-depleted cells exhibited a reduced expression of RhoA, ROCK1, and LIMK1. Zhao et al. provided compelling evidence that the silencing of B7-H3 diminished the migration and invasion capabilities of CRC cells mediated through the RhoA/ROCK1/LIMK1 signaling axis [93]. Furthermore, Chen et al. proposed a combined therapeutic approach; they discovered that B7-H3 stabilizes EGFR, thereby mediating cancer cell proliferation, accelerating tumor growth, and contributing to resistance to oxaliplatin (OXP). In colorectal cancer (CRC) tissues that expressed B7-H3, there were increased levels of EGFR, EGF, and PCNA, along with elevated markers of epithelial–mesenchymal transition (EMT). Conversely, silencing B7-H3 resulted in a reduction in EGFR and pERK levels. These findings suggest that B7-H3 may enhance the metastatic potential of cancer cells in CRC through the ERK/EGFR pathway. Targeting both B7-H3 and EGFR could improve responses to OXP chemotherapy both in vitro and in vivo, indicating that CRC patients might benefit from a dual blockade of B7-H3 and EGFR [94].

### 5.2. Metabolism and Angiogenesis Regulation

B7-H3 was also demonstrated to modulate glucose metabolism in cancer cells. In esophageal squamous cell carcinoma (ESCC), B7-H3 enhanced glucose turnover through the phosphorylation of pyruvate kinase M2 (PKM2) and the activation of STAT3 signaling pathways [95]. Similarly, in breast and colorectal cancers, B7-H3 exerts comparable effects by inhibiting Nrf2, thereby regulating critical metabolic enzymes such as hypoxia-inducible factor 1-alpha (HIF1α), lactate dehydrogenase A (LDHA), and pyruvate dehydrogenase kinase 1 (PDK1) [96]. In gastric cancer, mesenchymal stem cells (GCMSCs) have been implicated in inducing chemoresistance to oxaliplatin (OXA) and Paclitaxel (PTX) through the regulation of hexokinase 2 (HK2), possibly involving the TNF-α/p38 signaling pathway, with B7-H3 silencing mitigating this effect [97]. Additionally, Jin et al. demonstrated that B7-H3 inhibits cholesterol metabolism in CRC, both in vitro and in vivo. Analyses of differentially expressed genes (DEGs) in cells with reduced B7-H3 expression indicated its potential role in cholesterol homeostasis and biosynthesis. Notably, B7-H3 knockdown resulted in increased levels of total cholesterol and LDL-C, as well as the enhanced expression of cholesterol metabolism-related genes. Furthermore, B7-H3 downregulated sterol regulatory element binding protein 2 (SREBP2) through the activation of AKT [98].

Angiogenesis is another process potentially influenced by B7-H3. It has been shown to promote angiogenesis in tumors via diverse signaling pathways, including AKT1/mTOR/VEGFA, PI3K/AKT/MMPs, NF-κB activation, and the Tie-2 pathway. Recently, Hu and colleagues demonstrated that B7-H3 regulates HB-EGF levels, impacting HIF-1α activity through the PI3K-AKT pathway. Their research revealed that B7-H3 is involved in protein phosphorylation and vascular development. B7-H3 knockdown decreased HIF-1α binding to the promoter region of HB-EGF and downregulated the PI3K/AKT/mTOR pathway proteins. Additionally, targeting CD276 reduced cancer cell proliferation, migration, and angiogenic abilities in endothelial tube formation assays. Silencing B7-H3 also led to the downregulation of HB-EGF, resulting in decreased tumor growth in CRC cell lines and cancer tissues [99].

### 5.3. Apoptosis Inhibition

In addition, CD276 has been shown to transduce anti-apoptotic signals in tumor cells, thereby contributing to accelerated tumor growth and tumorigenesis. In colorectal cancer (CRC), B7-H3 has been found to promote Jak2-STAT3 signaling, which increases the expression of the anti-apoptotic proteins Bcl-2 and Bcl-xl. Furthermore, CD276 inhibits cellular senescence in CRC by activating the AKT/TM4SF1/SIRT1 pathway [100]. Recent studies have confirmed the role of B7-H3 in inhibiting apoptosis across various malignancies and through multiple signaling pathways. Sun et al. demonstrated that B7-H3 could exert this effect via interactions with fibronectin and PI3K/AKT signaling in gastric cancer. The addition of fibronectin significantly reduced early cell apoptosis in the control group exhibiting high B7-H3 expression, but not in cells with reduced B7-H3 levels. Similarly, the levels of apoptotic proteins (including caspase 8, caspase 9, Apaf1, and cleaved PARP) decreased following fibronectin treatment, but only in B7-H3-high cells. Furthermore, the addition of fibronectin resulted in the increased phosphorylation of PI3K and AKT, as well as elevated Bcl-2 levels, accompanied by decreases in p53, Bax, and caspase 3 exclusively in cells expressing B7-H3. Conversely, silencing B7-H3 led to an upregulation of apoptotic proteins p53 and caspase 3 [92]. Additionally, B7-H3 may play a role in ferroptosis, a regulated form of cell death associated with intracellular iron accumulation. Analysis of ferroptosis-related gene expression (PTGS2, FTL, FTH, and GPX4) indicated that B7-H3 overexpression increased CRC cell resistance to ferroptosis, whereas silencing B7-H3 had the opposite effect. Notably, supplementation with exogenous cholesterol or treatment with botulin, an SREBP2 inhibitor, could overcome the B7-H3-mediated inhibition of cholesterol metabolism and ferroptosis resistance in CRC cells [48]. Thus, B7-H3 could serve as a ferroptosis regulator by modulating cholesterol metabolism in CRC.

Recently, Yamato and colleagues highlighted the clinical potential of targeting B7-H3 through a novel antibody–drug conjugate that incorporates a DNA topoisomerase I inhibitor, DS-7300a. Treatment with DS-7300a led to an increase in the levels of cleaved PARP and phosphorylated checkpoint kinase 1, indicating enhanced apoptosis in human cancer cell lines as well as in xenograft mouse models [101]. Additionally, another anti-B7-H3 drug conjugate, ITC-6102RO, was found to reduce cell viability and promote apoptosis by inducing DNA damage and causing cell cycle arrest in the S phase in lung and breast cancer cell lines [102]. These findings suggest that targeting B7-H3 may serve as a promising strategy in cancer therapy, producing anti-tumor effects through mechanisms that are independent of the patient’s immune response. However, further studies are necessary to fully understand the impact of B7-H3 blockade on human tumors.

## 6. B7-H3 in Gastrointestinal Tumors

### 6.1. Colorectal Cancer (CRC)

#### 6.1.1. B7-H3 Expression in CRC

Numerous researchers have demonstrated that B7-H3 is significantly overexpressed in CRC compared to healthy tissues or benign diseases [79,103]. The expression rates of B7-H3 in tumors vary across different studies. For instance, Wu et al. reported that 87.6% (197 out of 225) of CRC cases were B7-H3 positive [104]. In a study involving 805 CRC patients, Lu and colleagues identified weak B7-H3 staining in 30.9% (249 out of 805) of the cases, medium staining in 12.4% (100 out of 805), and strong staining in 7.6% (61 out of 805) [67]. Moreover, the expression of B7-H3 in primary tumors was correlated with its expression in corresponding metastases. The presence of CD276 was primarily found in tumor cells or vascular endothelial cells and was not detected in the immune cells of the tumor microenvironment in CRC [72]. Additionally, Kovaleva and colleagues reported that median soluble B7-H3 (sB7-H3) levels were significantly elevated in the blood of CRC patients compared to healthy donors, suggesting its potential role as a disease marker [11]. Furthermore, the increased expression of B7-H3 in CRC as opposed to healthy tissue may provide a promising basis for developing novel, low-toxic therapeutic strategies. Recently, Zekri et al. proposed T cell-recruiting B7-H3xCD3 bispecific antibodies as an effective means of stimulating T cells in CRC while minimizing off-target T-cell activation [105].

#### 6.1.2. B7-H3 Influence on Clinicopathological Characteristics, Immune Responses, and Tumorigenesis in CRC

The upregulation of B7-H3 in CRC is closely associated with more advanced tumors and a more aggressive disease phenotype, which includes a higher TNM stage, a larger tumor size, and the presence of lymph node metastasis [98]. Interestingly, Kovaleva et al. found a correlation between higher sB7-H3 levels and the absence of regional metastasis, while sB7-H3 was linked to tumor progression [11]. This finding contrasts with another report that showed elevated serum B7-H3 levels correlated with a more severe TNM stage and metastasis [103]. Moreover, most authors have identified a negative correlation between B7-H3 expression in CRC tissues and tumor-infiltrating lymphocytes (TILs) [79,106]. Increased CD276 levels were associated with reduced CD8 T-cell infiltrations and a decrease in CD4 memory T-cells [72]. Conversely, B7-H3 upregulation was associated with an increased infiltration of monocytes and macrophages, particularly M2 macrophages, as well as heightened numbers of Tregs, eosinophils, and neutrophils [80]. These results indicate that excessive B7-H3 expression in CRC alters the immunological landscape of the TME, leaning toward promoting immunosuppressive activity. Higher B7-H3 expression in CRC was correlated with poorer survival outcomes, as reported by various studies. In the TCGA COAD cohort, high B7-H3 expression in patients was linked to shorter overall survival (OS) [107]. In contrast, Kovaleva et al. demonstrated that the presence of sB7-H3 in the blood of CRC patients corresponded to a favorable prognosis [11]. The authors proposed that tumorigenesis might be associated with increased sB7-H3 secretion, but cancer progression leads to decreased sB7-H3 levels. Overall, B7-H3 upregulation appears to relate to a more aggressive disease course and less favorable patient outcomes. Despite earlier reports suggesting an anti-cancer role for B7-H3, recent studies indicate that B7-H3 has several protumor effects, playing a significant role in tumorigenesis and the progression of CRC [108]. According to Peuker and colleagues, abnormal B7-H3 expression, induced indirectly by myeloid calcineurin–NFAT pathway activation due to microbiota, resulted in inhibiting CD8 T-cells [72]. Furthermore, B7-H3 upregulation increased the production of Th1/Th2 cytokines (including TNF-α, IL-2, IL-4, IFN-γ, IL-6, and IL-10) in the TME and triggered TNF-α secretion in CRC cells, contributing to tumor growth. B7-H3 may also enhance glucose metabolism in cancer cells by promoting the expression of hexokinase 2 (HK2). Additionally, B7-H3 increased resistance to ferroptosis in CRC cells by modulating cholesterol metabolism through downregulating SREBP2 and activating AKT signaling [71]. Moreover, the overexpression of B7-H3 in CRC promoted cancer cell proliferation, migration, invasion, and angiogenesis. These effects are likely mediated through B7-H3-dependent activation of JAK2-STAT3, NF-κB/VEGFA, HIF-1α, and AKT1/mTOR/VEGFA signaling pathways, as previously discussed [35,98].

#### 6.1.3. Possible Therapeutic Approaches Involving B7-H3

Recent reports indicate that inhibiting the RhoA/ROCK1/LIMK1 pathway, which is involved in the CD276-dependent regulation of the actin cytoskeleton, can reverse the effects of B7-H3 overexpression on the aggressive phenotype of CRC cells [93]. Targeting B7-H3 and its interacting partners could serve as an effective anti-cancer strategy, potentially decreasing the aggressiveness of the disease. Additionally, B7-H3 has been shown to mediate chemoresistance in CRC. Specifically, CD276, through STAT3 signaling, can elevate the expression of CDC25A, contributing to resistance against oxaliplatin (L-OHP). Furthermore, CD276 inhibits cancer cell responses to doxorubicin (DOX) via the AKT/TM4SF1/SIRT1 pathway, while knocking down B7-H3 promotes cell senescence upon DOX treatment [100]. The enhanced expression of B7-H3 in CRC also reduces the effectiveness of radiotherapy on the cells through the B7-H3/KIF15/ERK axis. Notably, CRC cells express CD276 more abundantly following radiation exposure. Blocking B7-H3 leads to the increased sensitivity of CRC cells to irradiation in vivo [109]. These findings suggest that incorporating anti-CD276 strategies into CRC treatment could enhance patient responses to existing therapies. Moreover, B7-H3 could act as a potential marker for predicting patient reactions to treatment. Therefore, future research should aim to determine the effects of B7-H3 blockade in CRC patients and explore the prognostic potential of measuring soluble B7-H3 (sB7-H3) levels.

#### 6.1.4. The Clinical Significance of ICIs for CRC Treatment

In CRC, immune checkpoint inhibitors (ICIs) targeting the PD-1/PD-L1 axis have gained approval for use exclusively in tumors exhibiting mismatch repair deficiency (MMR-D) and high microsatellite instability (MSI-H), which are associated with mutations in DNA mismatch repair (MMR) genes, present in approximately 15% of CRC cases [110]. Recent research suggests that MSI/MSS status may not serve as a definitive biomarker for predicting response to immune checkpoint inhibition, as it is not strictly correlated with immune infiltration or the expression of immune checkpoints. Additional genetic factors, such as the presence of POLE mutations, may also influence immune scores independently of MSI/MSS status, thereby altering the immune phenotypes of microsatellite-stable (MSS) tumors, making them similar to MSI tumors. Studies evaluating the expression of immune checkpoints, including B7H3 in CRC, have frequently not analyzed clinically relevant mutations such as BRAF and KRAS [111]. The BRAF oncogene encodes a serine/threonine kinase that, when activated, induces the expression of genes responsible for cell proliferation and survival. BRAF mutations are found in more than 50% of MSI tumors and around 10% of MSS tumors. Regardless of MSI/MSS status, tumors harboring BRAF mutations exhibit increased immune cell infiltration and a higher expression of immune checkpoints [111]. KRAS, another oncogene, is implicated in uncontrolled cell proliferation and is one of the most prevalent driver mutations in CRC, present in 30–40% of tumors [112]. Emerging evidence suggests that KRAS mutations correlate with a compromised immune landscape and the inhibition of immune pathways [113]. Activating mutations in PIK3CA, a catalytic subunit of PI3K, are found in 10–20% of all CRCs and lead to the uncontrolled activation of the AKT and mTOR pathways. The most frequent localizations of over 80% of mutations are in two hot spots in exon 9 and exon 20 [114]. The presence of PIK3CA mutations is associated with elevated PD-L1 expression and increased immune infiltration in CRC, while in tumors devoid of RAS mutations, these mutations correlate with poor clinical outcomes and resistance to anti-EGFR therapy [115]. Finally, alterations in the AKT1 gene, a serine–threonine kinase within the PI3K signaling pathway, promote tumor growth and inhibit apoptosis through unregulated activation [116].

Given the limited effectiveness of currently registered immunotherapeutic agents in a small subset of colorectal cancer (CRC) patients, and the insufficiently explored predictors of therapeutic responsiveness, identifying novel immune targets and biomarkers for optimal patient selection is paramount. In our previous paper, we found that the positive expression of B7H3 in CRC tumors was not associated with MSI/MSS status [79].

### 6.2. Esophageal Cancer

#### 6.2.1. B7-H3 Expression in EC

B7-H3 is upregulated in esophageal cancer (EC) compared to healthy esophageal tissues [81,117]. In EC, B7-H3 is primarily located in the cell membrane and cytoplasm of cancer cells. Wang and colleagues also observed that B7-H3 was co-expressed with B7-H4 in 47 out of 66 (71.2%) esophageal squamous cell carcinoma (ESCC) samples [81]. A high expression of B7-H3 is associated with a more advanced TNM stage, increased tumor invasion depth, and a more advanced clinical stage [76,117]. Xu and colleagues demonstrated that patients with lymph node metastasis had elevated B7-H3 mRNA levels in tumors [118]. However, Wang et al. reported a link between the absence of lymph node metastasis and increased B7-H3 expression [81]. These contradictory results may stem from small sample sizes and a limited number of studies examining the correlations between B7-H3 expression in EC and the clinicopathological features of patients. Recently, Xiong and colleagues discovered higher B7-H3 expression in a recurrent group of ESCC patients [76]. Additionally, lower B7-H3 levels were noted in patients with a better response to first-line therapy [76]. Interestingly, one study found that B7-H3 expression was higher in females (*p* = 0.0229) [117], while another study by Song et al. reported increased levels in patients over 60 years old, although these findings have not been consistently confirmed in other studies [119]. Overall, higher B7-H3 expression in esophageal cancer appears to correlate with a more aggressive disease, but the existing literature on this topic is relatively limited. Several authors have concluded that EC patients with high B7-H3 expression in tumors have significantly worse overall survival (OS) and progression-free survival (PFS) compared to those with low B7-H3 expression [81,82]. Furthermore, the combined elevated expression of B7-H3 and B7-H4 was associated with poorer outcomes than other expression combinations [81,117]. These findings suggest that B7-H3 immunolabeling could serve as a prognostic factor for the disease. Additionally, therapies targeting both B7-H3 and B7-H4 may provide greater therapeutic benefits, but further investigation is necessary. The current findings regarding B7-H3’s role in esophageal malignancies support its immunoinhibitory functions.

#### 6.2.2. Influence on Immunity and Therapeutic Options

Wang and colleagues reported a positive association between B7-H3 and infiltrates of regulatory T cells (Treg) (*p*  =  0.003) and tumor-associated macrophages (TAMs) (*p*  =  0.021), along with a negative correlation with CD8+ T cells [81]. Additionally, Chen et al. found a negative correlation between B7-H3 levels and CD3+ T cells [82]. These results suggest that increased B7-H3 expression may suppress T cell anti-tumor responses and facilitate immune escape in esophageal cancer. Various effects of B7-H3 knockdown (KD) in EC cells have been documented. Silencing B7-H3 resulted in reduced cancer cell migration and invasion [82]. Chen et al. demonstrated that B7-H3 KD suppressed cancer cell proliferation [82], a finding confirmed for ESCC cells obtained from mouse models by Xiong et al. [76]. Xiong et al. also showed that B7-H3 silencing in mice led to reduced tumor size and fewer lesions compared to controls. Cancer cells from B7-H3 knockout (KO) mice exhibited increased apoptosis, while their tumors displayed enhanced CD8+ T cell infiltration and a higher number of natural killer (NK) cells, along with reduced neutrophil counts. The authors suggested that B7-H3 promotes neutrophil extracellular trap (NET) formation through the CXCL1/CXCR2 axis, which leads to NK cell suppression and contributes to protumor effects in ESCC. Therefore, B7-H3 depletion may exert anti-tumor effects primarily by enhancing NK cell activity [76]. More studies are needed to confirm this mechanism and its relevance for EC patients. Nevertheless, these findings highlight the potential for B7-H3 targeting to reduce the aggressiveness of the disease and enhance immune responses in esophageal malignancies. Recently, new therapeutic strategies targeting B7-H3 have been proposed for EC. Wang et al. introduced a tumor microenvironment (TME)-regulated chimeric antigen receptor T (MRS.CAR-T) system, featuring an MMP cleavage site and an HSA-binding peptide, which activates only in a solid tumor microenvironment. This approach could mitigate the potential cytotoxic effects of B7-H3 targeting associated with its high physiological expression in organs such as the adrenal gland, pancreas, gallbladder, prostate, ovary, and cervix while effectively eliminating EC cells and inducing T memory cells [120]. Wu and colleagues presented anti-B7-H3 monoclonal antibodies (mAbs) that demonstrated strong activity against esophageal squamous cell carcinoma (ESCC). In their study using a patient-derived xenograft (PDX) model of ESCC, the group observed that the tumors in the mAb-treated cohorts had lower volumes and weights, along with an increased infiltration of natural killer (NK) cells [121]. These findings highlight the clinical potential of B7-H3 and underscore the importance of further exploring its role in the treatment of esophageal cancer.

### 6.3. Gastric Cancer

#### 6.3.1. B7-H3 Expression in GC

B7-H3 is notably overexpressed in gastric cancer (GC) compared to surrounding healthy tissue [44,122]. Ulase et al. found that among 96 gastric cancer samples, 41 exhibited moderate to strong B7-H3 staining. This molecule was predominantly expressed in the stromal compartment, observed in 76% of cases, but its presence was also noted in cancer cells [123]. Other studies have reported B7-H3 expression in immune cells within the tumor microenvironment (TME), including CD68-positive macrophages and intratumor neutrophils, as well as in α-SMA-positive fibroblasts [83,124]. Additionally, Arigami and colleagues found significantly higher B7-H3 mRNA levels in the blood of gastric cancer patients compared to healthy individuals (*p* < 0.0001) [125]. Elevated B7-H3 expression was also noted in patients with H. pylori infection. There is a link between higher B7-H3 expression in tumors and more advanced tumor stages, tumor depth, and lymph node involvement [44]. Ulase et al. revealed a correlation between high B7-H3 levels in stromal cells and both proximal stomach tumor location and Laurén phenotype [123]. Furthermore, B7-H3 upregulation was associated with vascular infiltration (*p* = 0.01), perineural invasion (*p* < 0.01), and more advanced TNM stages (*p* < 0.01) in another study [126]. Guo and colleagues found that B7-H3 expression increased with cancer progression, being barely detectable during the inflammatory phase and showing positive staining in 78% (39/50) of gastric adenocarcinoma specimens [83]. Similarly, higher B7-H3 mRNA levels in blood were correlated with more advanced stages of gastric cancer, indicating its potential as a useful blood marker [125]. Interestingly, the co-expression of B7-H3 and CD47 in cancer cells was associated with larger tumors and greater invasiveness compared to tumors without such co-expression [84]. CD47, an integrin-associated protein, is known as an antiphagocytic molecule linked to a worse prognosis in cancer patients [84]. Multiple studies have found a correlation between elevated B7-H3 expression in gastric cancer and poorer patient survival. The high co-expression of B7-H3 with HER2 was identified as an independent risk factor for poorer overall survival (OS), and CD47 co-expression was associated with worse outcomes than the expression of either B7-H3 or CD47 alone in tumor samples (*p* = 0.0007) [84].

#### 6.3.2. B7-H3 Influence on Immune Responses in GC

B7-H3 also influences the immunological landscape in gastric tumors. Guo et al. showed that B7-H3 expression in cancer cells negatively correlated with the number of CD8+ T cells, while in immune cells, there was a positive correlation with CD68-expressing cells, suggesting an increase in tumor-associated macrophage (TAM) infiltration [84]. Furthermore, Chen and colleagues found a positive correlation between B7-H3 expression and markers characteristic of common/M2 macrophages (CD68+/CD163+) [84]. Notably, in B7-H3-high tumors, T lymphocytes were less frequently localized and primarily accumulated at the tumor invasive front (IF). The elevated expression of B7-H3 led to the suppression of CD8+ T cell activity in the tumor center, indicating that B7-H3 upregulation in gastric cancer may promote TAM abundance and inhibit T-cell responses [123]. Furthermore, in advanced gastric cancer patients, a higher number of intratumor B7-H3+ neutrophils was observed compared to those with early-stage disease. Li et al. demonstrated that increased neutrophil infiltration in GC is associated with poor survival. Consistently, patients with high percentages of intratumor B7-H3+ neutrophils exhibited a lower 25-month survival rate [85]. These findings indicate that immune responses in gastric cancer are adversely affected by B7-H3 overexpression in cancer cells or the TME, resulting in worse patient outcomes.

#### 6.3.3. Potential Therapeutic Options

In gastric cancer cell lines, silencing B7-H3 resulted in reduced cell migration and invasion [44]. Moreover, B7-H3 knockdown diminished the number, size, volume, and weight of tumors in xenograft mouse models. Finally, the loss of B7-H3 decreased the migration and invasion of cancer-associated fibroblasts (CAFs) [124]. Importantly, targeting B7-H3 may also enhance gastric cancer patients’ sensitivity to radiotherapy. Li and colleagues demonstrated that B7-H3 contributes to the radioresistance of gastric cancer cells by modulating their autophagy and decreasing DNA double-strand breaks. In vivo studies showed that silencing B7-H3 inhibited cancer cell growth post-radiation [127].

Several researchers have proposed novel therapeutic strategies for GC that involve targeting B7-H3. Sun et al. introduced CAR-T cells specifically targeting B7-H3 (CAR.B7-H3-T), which demonstrated strong cytotoxicity toward GC cells with high B7-H3 expression while showing no toxicity to B7-H3-negative cells. Notably, CAR.B7-H3-T cells were also effective in killing cancer stem cells (CSCs) with an efficacy similar to that seen in GC cells. In mouse models, CAR.B7-H3-T cells efficiently eliminated tumor cells, inhibited tumor growth, and enhanced mouse survival without causing noticeable toxicity in vital organs [122]. Lutz and colleagues proposed a novel bispecific antibody, known as CC-3, which is based on an IgG format and has specificity for both B7-H3 and CD3. CC-3 exhibited high cytotoxicity against GC cells and effectively induced T-cell activity in the presence of B7-H3-positive cells [128].

### 6.4. Hepatocellular Carcinoma and Cholangiocarcinoma

#### 6.4.1. B7-H3 Expression in Hepatocellular Carcinoma and Cholangiocarcinoma

B7-H3 was found to be upregulated in hepatocellular carcinoma (HCC) compared to normal liver tissues and benign hepatic hemangioma tissues [87]. Zhao and colleagues found that soluble B7-H3 (sB7-H3) was elevated in the sera of patients with early-stage hepatocellular carcinoma (ESHCC) compared to cirrhotic patients (*p* < 0.001). This suggests that sB7-H3 could serve as a useful marker for differentiating between these two conditions, with a higher area under the curve (AUC) value of 0.898 compared to AFP, CA125, or CA199 [129]. In another study, sB7-H3 levels proved significant for HCC diagnosis, reaching an AUC value of 83.2%. Moreover, measuring sB7-H3 alongside IL-17, IL-8, and IL-6 concentrations was even more effective for detecting the disease, as these markers showed an AUC value of 99.2%, a sensitivity of 96.67%, and a specificity of 97.14% [130]. In a study involving 116 HCC patients, increased B7-H3 expression in HCC tissues correlated with a more advanced TNM stage, vascular invasion, the presence of metastases and lymph node metastasis, larger tumor size, and microsatellite tumor formation [131]. Higher B7-H3 expression was also linked to recurrent disease and shorter survival of patients [131]. Similarly, elevated sB7-H3 in serum was associated with a more advanced TNM stage, larger tumor size, vascular invasion, poor tumor differentiation, and shorter median survival time (MST) [129].

Similar trends were observed for intrahepatic cholangiocarcinoma (ICC). The positive expression of B7-H3 was linked to increased disease aggressiveness, as indicated by the presence of lymph node metastases and venous invasion. Additionally, a positive correlation was found between B7-H3 expression and microvessel density in tumors. Overall survival (OS) and cancer-specific survival (CSS) were significantly shorter in cases with the upregulation of B7-H3, making it an independent prognostic factor for poor OS and CSS (*p* = 0.002). Cheng et al. have suggested that B7-H3 could serve as a valuable biomarker and a target for antivascular therapy in ICC; however, further studies are needed to confirm these findings [87].

#### 6.4.2. Effect on Immunity and TME

In HCC, B7-H3 has been identified as a factor influencing immune infiltrations within the tumor microenvironment (TME). The levels of B7-H3 correlated positively with tumor-associated macrophages (TAMs) and negatively with CD8+ T cell infiltration [91]. B7H3 promoted the differentiation of M2 macrophages and increased mRNA levels of VEGF and macrophage-derived chemokine (CCL22) through STAT3 signaling activation [86]. Moreover, silencing B7-H3 led to the increased secretion of IFNγ and TNFα, as well as enhanced cytotoxicity of CD8+ T cells against HCC cells [132]. In a murine model of HCC, B7-H3-targeting antibody treatment resulted in reduced tumor size and increased anti-tumor T-cell responses, which prolonged mouse survival [132]. Furthermore, B7-H3 was found to enhance cancer cell proliferation, adhesion, migration, and invasion [87]. Kang and colleagues also noted that B7-H3 promoted epithelial-to-mesenchymal transition (EMT) by activating the JAK2/Stat3/Slug signaling pathway [86]. Interestingly, EMT induced by TNF-α or TGF-β1 could also lead to the upregulation of B7-H3 in HCC cells [133]. Conversely, the knockdown of B7-H3 reversed TGF-β1-induced EMT and decreased HCC cell migration in transwell assays [134]. These findings indicate that targeting B7-H3 may serve as a potent anti-tumor strategy for HCC by enhancing anti-tumor immune responses and reducing disease aggressiveness.

Recently, Cao et al. proposed a novel chimeric antigen receptor T (CAR-T) cell therapy strategy using bispecific T cell engagers (CAR.T-BiTEs) that target both B7-H3 and Glypican-3 (GPC3). This approach increased T-cell activity and cytotoxicity against B7-H3-positive HCC cells, resulting in the superior elimination of these cells compared to therapies targeting GPC3 alone or B7-H3 alone. This strategy may address the issue of antigen escape, which has been a challenge for GPC3 CAR-T cells due to the heterogeneous expression of GPC3 in HCC [135].

### 6.5. Pancreatic Cancer

#### 6.5.1. B7-H3 Expression in Pancreatic Cancer

Several authors have reported an abundant expression of B7-H3 in pancreatic cancer compared to normal pancreatic tissues. In a study involving 150 patients with pancreatic ductal adenocarcinoma, Inamura et al. found B7-H3 expression in cancer cells in 99 samples (66%) [136]. In contrast, Geerdes and colleagues detected B7-H3 expression in tumor cells in only 29 out of 137 samples (21%) [137]. While B7-H3 levels were higher in the tumor stroma, a strong expression of CD276 was not observed in pancreatic cancer cells [137]. Other studies have also shown the presence of B7-H3 in tumor-associated macrophages (TAMs) and tumor-associated vasculature (TAV) [66,138]. Various studies indicate no significant association between B7-H3 expression in pancreatic adenocarcinoma (PAAD) and most clinicopathological features, including the TNM stage. However, higher B7-H3 levels were observed in poorly differentiated PAAD compared to well-differentiated tumors [89]. Cattaneo et al. also reported a positive association between B7-H3 and HLA class I expression [66]. Conversely, in PanNETs, B7-H3 expression was linked to a more aggressive disease course: the presence of metastases, an elevated Ki67 proliferation index, a higher mitotic rate, an advanced WHO grade, and lymphovascular or perineural invasion [139]. Similarly, a higher infiltration of B7-H3+ macrophages in pancreatic neuroendocrine tumors was associated with lymph node involvement, higher histopathological grades, more advanced TNM stages, and the presence of metastasis [140]. Further studies are needed to clarify the relationship between CD276 and pancreatic cancer progression. Nevertheless, B7-H3 could be a promising target for therapies against PanNETs, warranting future research in this area.

#### 6.5.2. B7-H3 Influence on Prognosis and Immune Responses in Pancreatic Cancer

The associations between B7-H3 and survival in pancreatic cancer remain unclear. Loos and colleagues found that high B7-H3 expression was associated with better overall survival (OS) rates (*p* = 0.0067) in pancreatic cancer patients [88]. In contrast, Inamura et al. reported that elevated B7-H3 expression corresponded to lower disease-free survival (DFS), particularly in the earlier stages of the disease, suggesting that the molecule may serve as a useful biomarker in early-stage pancreatic cancer [136]. Moreover, according to Cattaneo et al., elevated B7-H3 corresponded to poor survival only in patients with proper HLA class I expression, possibly due to severely impaired immune anti-cancer responses in the absence of HLA class I. Considering that in patients with HLA class I present, the survival was better in the event of low B7-H3 expression, targeting B7-H3 could be a promising therapeutic strategy for patients with high HLA class I pancreatic cancer [66]. Additionally, positive B7-H3 expression in tumor stroma was associated with poorer progression-free survival (PFS), and a higher infiltration of B7-H3+ macrophages was linked to worse survival in pancreatic neuroendocrine neoplasms [139]. Conflicting reports also exist regarding the correlations between B7-H3 and the immunological landscape in PAAD. Loos et al. found that B7-H3 was positively associated with CD8 T-cell infiltrations and IFN-γ expression, suggesting that the molecule may stimulate anti-tumor immune responses [88]. Additionally, Si and colleagues discovered that CD276 was positively correlated with CD8+ T cells, CD4+ T cells, neutrophils, macrophages, and dendritic cells (DCs). On the contrary, B7-H3 and B7-H4 co-deficiency predicted immune-hot tumors with high CD8 TILs [89].

Currently, little information is available regarding the regulation of B7-H3 expression in pancreatic tumors. In Cattaneo et al.’s study, B7-H3 expression could be promoted similarly to HLA class I transcription through the NF-κB subunit p65 (RELA) [66]. Furthermore, Zhao and colleagues discovered that the histone acetyltransferase (HAT) BRD4 enhanced B7-H3 expression, leading to Toll-like receptor 4 (TLR4) upregulation. The authors suggested that targeting the BRD4/B7-H3/TLR4 pathway could represent a novel strategy for overcoming immunotherapy or chemotherapy resistance [46]. In neuroendocrine tumors, B7-H3 expression on macrophages was induced by PIWI-interacting RNA (piRNA) piR-hsa-30937 in small extracellular vesicles (sEVs) derived from pancreatic neuroendocrine neoplasms (PNENs). These sEVs containing piR-hsa-30937 were internalized by macrophages, which resulted in the downregulation of PTEN (phosphatase and tensin homolog deleted on chromosome 10) and the subsequent activation of AKT signaling, leading to B7-H3 upregulation. B7-H3+ macrophages facilitated the proliferation and metastasis of PNENs in mouse models by inhibiting T-cells, and blocking B7-H3 reversed these effects [140]. However, limited data are available regarding the outcomes of B7-H3 silencing in pancreatic cancer, and existing reports present conflicting suggestions. More research is needed to verify the function and clinical potential of this molecule in pancreatic tumors.

#### 6.5.3. Regulation of B7-H3 Expression in Pancreatic Tumors and Potential Therapeutic Strategies

Several authors have proposed B7-H3-targeting anti-cancer strategies for pancreatic tumors. Lutz et al. introduced CC-3, a bispecific antibody (bsAb) that targets B7-H3 and CD3, which activated T-cell responses against pancreatic cancer cells. CC-3 increased the secretion of IL-2, IFN-γ, IL-10, and TNF, stimulated the proliferation of effector memory and central memory T cells in the presence of B7-H3-positive cells, and significantly destroyed pancreatic cancer cells [128]. In another study, the authors proposed a monoclonal antibody targeting B7-H3 (B7-H3-SDIE). B7-H3-SDIE enhanced the activation of various natural killer (NK) cell subsets, induced IFN-γ and TNF secretion in the presence of B7-H3-expressing cells, and led to the effective lysis of pancreatic cancer cells [141]. Additionally, Wang and colleagues demonstrated that irradiation caused increased B7-H3 expression in pancreatic cancer cell lines, which enhanced the ability of B7-H3 CAR-T cells to kill tumor cells. The combination of CAR-T therapy and radiotherapy further improved CAR-T cell activity on non-irradiated tumor areas, illustrating an abscopal effect [142]. In conclusion, these findings indicate that B7-H3-targeting strategies could be effective against pancreatic malignancies. However, the potential off-target effects and the influence of B7-H3 blockade on other cell populations still need to be verified.

The expression patterns of B7 family members (B7H3, B7H4, HHLA2, and PD-L1) are presented in Table 2.

## 7. Conclusions and Perspectives

Recent advancements in the treatment of solid tumors have markedly improved patient prognosis, fostering optimism for the potential transformation of cancers into manageable chronic diseases. The rapid evolution of immunotherapy, particularly the deployment of immune checkpoint inhibitors (ICIs), has emerged as a cornerstone of contemporary cancer therapeutics, guiding new paradigms for personalized treatment strategies. However, a significant subset of patients remains refractory to ICIs or develops resistance during the course of therapy. Additionally, the application of ICIs poses the risk of immune-related toxicities, which can lead to severe organ dysfunction and necessitate the discontinuation of treatment.

Certain malignancies demonstrate a lack of response to immune checkpoint blockade, primarily attributed to the low expression of immune checkpoints targeted by current immunotherapeutic agents. Pembrolizumab, an immune checkpoint inhibitor that targets PD-L1, is approved solely for use in microsatellite instability-high (MSI-H) colorectal cancer (CRC), which represents about 15% of all CRC cases. In contrast, microsatellite-stable (MSS) tumors, which are present in the majority of patients, do not express PD-L1, rendering therapies aimed at this immune checkpoint ineffective [153]. Our previous research involving a limited cohort of CRC patients indicated that B7-H3 expression was not associated with MSI/MSS status; nonetheless, this finding necessitates validation through additional studies with larger patient populations, as research in this domain remains sparse [79].

The effectiveness of immunotherapy is intricately linked to the tumor immune landscape, which can be categorized into two predominant types: immune “hot” and immune “cold.” Immune-“hot” tumors are characterized by heightened immune infiltration, predominantly comprising CD8+ T lymphocytes, Th1 cells, M1 macrophages, and dendritic cells, along with an elevated expression of immune checkpoints, a high tumor mutational burden (TMB), and a substantial presence of neoantigens, which collectively enhance tumor recognition by the immune system. Additionally, the microenvironment of “hot” tumors displays increased levels of tumor-infiltrating lymphocytes (TILs) and augmented pro-inflammatory cytokine signaling with elevated concentrations of IFN-gamma, TNF-alpha, IL-2, and CXCL9, which promote T lymphocyte recruitment and activation within the immune tumor microenvironment (iTME) [6]. Tumors exhibiting an immune-“hot” phenotype include melanoma, MSI-H CRC, non-small-cell lung cancer (NSCLC), renal cell carcinoma (RCC), and triple-negative breast cancer (TNBC) [6]. These malignancies are more likely to respond favorably to immune checkpoint blockade, and clinical outcomes in this patient subset can be notably promising.

Conversely, a significant proportion of solid tumors are classified as immune “cold”, with a low expression of immune checkpoints, low TMB, and poor immune infiltration characterized by a scarcity of T cells, the absence of TILs, and a predominance of regulatory T cells (Tregs), M2 macrophages, and myeloid-derived suppressor cells (MDSCs). Such tumors are also marked by elevated levels of immunosuppressive cytokines, including TGF-beta and IL-10. The diminished presentation of antigens due to reduced MHC-I molecule expression, coupled with barriers to immune cell infiltration posed by stromal cells and tumor vasculature, culminates in the exclusion of T cells from the tumor microenvironment. Malignancies with an immune-“cold” phenotype—including MSS CRC, pancreatic cancer, glioblastoma, hepatocellular carcinoma (HCC), and most HR+/HER2- breast cancers—have been shown to be resistant to ICIs [6,154].

One of the foremost challenges in the therapy of solid tumors is the identification of immune targets expressed in the iTME of immune-“cold” tumors, along with the development of strategies aimed at converting the cold phenotype into a hot one. The increasing number of clinical trials investigating therapies targeting B7-H3 highlights the growing anticipation surrounding this relatively novel immune molecule. Its involvement in immune evasion from host surveillance, contribution to non-immunological processes that foster cancer promotion and progression, and cancer-specific expression profile have been corroborated by numerous studies.

Importantly, a comprehensive understanding of B7H3 receptors is essential, particularly regarding potential interactions with the TLT2 receptor, which could confer the costimulatory activities to B7H3. Such interactions may significantly impact the outcomes of B7H3-targeted inhibition aimed at promoting anti-tumor responses, as a blockade of TLT2 signaling could result in the opposite effects. Additionally, elucidating the co-expression patterns of B7H3 alongside other immune checkpoints, including PD-1/PD-L1, B7-H4, and HHLA2 in specific tumor types, is critical for the development of dual blockade immunotherapy approaches that are more precisely tailored and potentially more effective. Dual immune checkpoint inhibitors represent a leading strategy in the context of immune-cold solid tumors, leveraging the combined targeting of PD-1/PD-L1 and CTLA-4 pathways. For instance, the combination of ipilimumab, which targets CTLA-4, with nivolumab, an anti-PD-1 agent, has received approval for the treatment of melanoma, microsatellite instability-high (MSI-H) colorectal cancer (CRC), renal cell carcinoma (RCC), hepatocellular carcinoma (HCC), and PD-L1-positive non-small-cell lung cancer (NSCLC). Due to insufficient existing immunotherapeutic strategies, there is a great need to search for novel ICs with the potential for use in the treatment of gastrointestinal tumors. B7H3 could be a potent candidate in this venture. Recent studies demonstrate promising results of utilizing mAbs targeting B7H3 in ESCC and pancreatic cancer, CAR-T cells in gastric cancer, or CAR.T-BiTEs that target both B7-H3 and Glypican-3 in HCC. Still, the data regarding anti-B7H3 strategies in gastrointestinal tumors are limited, and the molecule’s potential should be verified in future works.

A significant challenge for current and future research is to clarify whether B7H3 expression delineates an immune signature characteristic of “hot” tumors akin to PD-L1 or if it is representative of the more modest immune landscape found in “cold” tumors. Recent studies suggest that B7H3 inhibition may represent a viable therapeutic option for patients with tumors that are unresponsive to the inhibition of the PD-1/PD-L1 axis, such as microsatellite-stable CRC. Finally, the restricted expression of B7H3 in cancerous tissues, coupled with its minimal expression in normal tissues, suggests a potentially favorable safety profile characterized by a reduced risk of severe immune-related adverse effects associated with B7H3 inhibition.

## Figures and Tables

**Table 1:** B7H3’s role in shaping the immune landscape in gastrointestinal tumors. In most malignancies discussed, B7H3 upregulation exerts immunosuppressive function, inhibiting T-cell and NK-cell activity, while increasing TAM, Treg, or neutrophil numbers.

	Influence of B7H3 Upregulation on the Immune Landscape		References
	Increase in the Following:	Decrease in the Following:	
Colorectal cancer	TAMs (M2 macrophages), Tregs, eosinophils, and neutrophils, Th1 scores	TILs: CD8 T-cells and CD4 memory T-cells, Th2 scores	[72,79,80]
Esophageal cancer	Tregs, TAMs, neutrophils	TILs/CD8+ T-cells, NK cells	[76,81,82]
Gastric cancer	TAMs (B7H3 in immune cells)/M2 macrophages, neutrophils	CD8 cells (B7H3 in cancer cells)	[83,84,85]
Hepatocellular carcinoma	TAMs, Tregs	CD8 T-cells	[86,87]
Pancreatic cancer	CD8 and CD4 T-cells, neutrophils, macrophages, DCs	-	[88,89]

**Table 2:** The expression of B7 family proteins (B7H3, HHLA2, B7H4, and PD-L1) in gastrointestinal tumors. CRC—colorectal cancer, GC—gastric cancer, HCC—hepatocellular carcinoma, PC—pancreatic cancer, EC—esophageal cancer, and “-“—no data available.

	B7H3			HHLA2			B7H4			PD-L1		
Tumor Type	Positive Rate	Cutoff for Positive Expression	Source	Expression Rate	Cutoff for Positive Expression	Source	Expression Rate	Cutoff for Positive Expression	Source	Expression Rate	Cutoff for Positive Expression	Source
CRC	32–87%	>1%, >10%	[79,80]	83.7%	>1%, >H score median	[143]	29.1–80%	>1%, H score > 85, final score > 3	[144]	9% *	>5%	[145]
GC	39.47–69.2%	median	[146,147]	53.2% (high expression)	final score ≥ 8	[143,148]	44.9–80%	staining 0, +/++, +++, final score > 2	[144,149]	11–69.4%	≥5%	[150]
HCC	70–93.75%	H-score ≥ 2	[17,147]	49.0–67.7%	IRS > 3, H-score ≥ 5	[143,148]	1–73%	-	[144,149]	24.06–34.5%	>1%, ≥20%, ≥75%	[151]
PC	41.18–77.78%	final score > 3	[147]	77%	H score > 80	[143,148]	22.1–76%	>0%, >1%, >10%	[144,149]	45%	≥10%	[152]
EC	55.75–69.7%	H-score > 185	[146,147]	-	-	-	53.8–95.5%	IHC score > 1, H score > 160	[144,149]	82.17%	score >0	[152]

* in CRC PD-L1 expression is limited to microsatellite-instable tumors, which comprise 15% of all CRC cases.

## Data Availability

Not applicable.
